# Usefulness of the hemogram as a measure of clinical and serological activity in systemic lupus erythematosus

**DOI:** 10.1016/j.jtauto.2022.100157

**Published:** 2022-05-13

**Authors:** Víctor Moreno-Torres, Raquel Castejón, Susana Mellor-Pita, Pablo Tutor-Ureta, Pedro Durán-del Campo, María Martínez-Urbistondo, José Vázquez-Comendador, Ángela Gutierrez-Rojas, Silvia Rosado, Juan A. Vargas-Nuñez

**Affiliations:** a Systemic Autoimmune Diseases Unit, Internal Medicine Service, IDIPHIM (University Hospital Puerta de Hierro Research Institute), Hospital Universitario Puerta de Hierro Majadahonda, Madrid, Spain; b Medicine Department, School of Medicine. Universidad Autónoma de Madrid, Madrid, Spain

**Keywords:** Red blood cell distribution width, Neutrophil-to-lymphocyte ratio, Platelet-to-lymphocyte ratio, IL-6, Systemic lupus erythematosus

## Abstract

**Background and objectives:**

Systemic Lupus Erythematosus (SLE) follow-up is based on clinical, and analytical parameters. We aimed to determine the differences between the Neutrophil-to-lymphocyte ratio (NLR), Platelet-to-lymphocyte ratio (PLR) and Red blood cell distribution width (RDW) between SLE patients and healthy controls and to assess their association with anemia status, classical inflammatory biomarkers and cytokines, disease activity, SLE related factors and treatment received for SLE.

**Methods:**

Seventy-seven patients with SLE according to 2012 SLICC criteria and 80 healthy controls were included. Patients with SLE were classified in SLE with anemia (SLE-a) and SLE without anemia (SLE-na). Statistical analysis between SLE patients and controls and the association of serological and clinical activity markers with proposed hematological indices among SLE patients were performed.

**Results:**

RDW, NLR and PLR, were significantly higher in SLE patients than in healthy control group (p < 0.001), in SLE-a patients as compared to SLE-na (p < 0.0001) and were significantly associated with hypocomplementemia (p < 0.05). PLR was higher in active patients measured by SLEDAI-2K score and with longer disease duration (p < 0.05). RDW was associated with serological activity of the patients (p < 0.05) and was correlated with SLEDAI-2K and SLICC/ACR scores, hsCRP, D-dimer, fibrinogen, IL-6 and TNF as well as with corticosteroids intake (p = 0.05). A logistic regression analysis confirmed that after adjustment by age and hemoglobin values, RDW presented linear correlation with IL-6 levels (Beta-coefficient = 0.369, p = 0.003).

**Conclusion:**

NLR, PLR and RDW values suggest SLE serological and clinical activity. Given their availability, these markers not only could be useful tools to identify and monitor active SLE patients but whose application should be considered in inflammatory pathologies orchestrated by IL-6 and TNF.

## Highlights

• RDW, NLR and PLR are higher in SLE patients than in the health patients.
• RDW correlates with SLEDAI-2k and SLICC/ACR scores, IL-6 and TNF.
• In anemic SLE patients, PLR reflects TNF activity.
• The value of these hematological indices supports SLE serological and clinical activity.

## Abbreviations

ANA: Antinuclear antibodies
AUC-ROC: Area Under the Curve - Receiver Operating Characteristic
*Anti*-dsDNA: anti-double-stranded DNA antibody
RA: Rheumatoid arthritis
DD: D-dimer
ESR: Erythrocyte sedimentation rate
hsCRP: High-sensitive C-reactive protein
IFNγ: Interferon γ
MCV: Mean corpuscular volume
MMF: mycophenolate mofetil
NLR: Neutrophil-to-lymphocyte ratio
PLR: Platelet-to-lymphocyte ratio
RDW: Red blood cell distribution width
SLE: Systemic lupus erythematosus
SLEDAI-2K: SLE Disease Activity Index
SLICC/ACR: Systemic Lupus International Collaborative Clinics/American College of Rheumatology
TNF: Tumor necrosis factor

## Funding (information that explains whether and by whom the research was supported)

This work has been supported by a grant from 10.13039/501100004587Instituto de Salud Carlos III (Expedient number PI16-01480): VMT has a grant from 10.13039/501100004587Instituto de Salud Carlos III (CM19/00223).

## 1 Introduction

Systemic lupus erythematosus (SLE) is a heterogeneous autoimmune disease that affects multiple organs, with a wide spectrum of clinical manifestations and characterized by a chronic systemic inflammation. Despite advances in diagnosis and treatment; follow-up, monitoring and prognosis of these patients is based mainly on clinical findings supported by serological and biochemical parameters, is very complex [1].

Haemogram and blood count are fundamental tools in the diagnosis and monitoring of these patients since, in addition to the anemia, lymphopenia or thrombopenia caused by the disease itself, many of the drugs used to control flares have hematological toxicity [2].

Recently, hematological indices such as the neutrophil-to-lymphocyte ratio (NLR) or platelet-to-lymphocyte ratio (PLR), which can be obtained easily using peripheral blood parameters, have been regarded as novel and accurate inflammatory biomarkers for predicting disease status and have already been considered in sepsis, neoplasms, inflammatory bowel disease and autoimmune or rheumatic diseases [3].

Similarly, red blood cell distribution width (RDW) is a parameter routinely reported as part of a complete blood count. RDW is calculated as the standard deviation of red blood cell volume divided by mean corpuscular volume (MCV), expressed as a percentage and it measures the size variability of circulating erythrocytes [4]. Traditionally, it has been considered in the differential diagnosis of iron deficiency anemia and anemia of chronic disease [5]. Inflammation has been suggested to lead to an elevation of RDW, ad there are multiple studies demonstrating that RDW levels correlate with inflammatory markers and disease activity in several chronic inflammatory diseases including cardiovascular disease, rheumatoid arthritis (RA), Sjögren's syndrome and Behçet disease [6]^.^

Some publications have also reported these cheaply and easily determined parameters and ratios as potential markers for inflammation and SLE activity [[7], [8], [9], [10], [11], [12]]. The aim of this study is to determine the differences between the NLR, PLR and RDW levels in SLE patients and healthy controls and to assess their association with anemia status, classical inflammatory biomarkers and cytokines, disease activity, SLE related factors as well to evaluate the treatment patients were receiving for SLE.

## 2 Methods

### 2.1 Study population, protocol, and clinical assessment

This cross-sectional study included 77 consecutively recruited female patients attending a scheduled appointment in the outpatients’ autoimmune diseases unit at our hospital who met 2012 SLICC criteria for the classification of SLE between January 1st, 2015 and December 31st, 2016 [[13], [23]]. Eighty healthy women haemograms (bone marrow donors between the ages of 18 and 64), obtained from our blood bank, were analyzed as a control group. The study was approved by the Research Ethics Committee of the Hospital Universitario Puerta de Hierro and written informed consent was obtained from all participants.

Patients were assessed for previous diseases, renal function, liver profile, comorbidities and SLE-related factor including antiphospholipid syndrome according to criteria [[14], [24]]. The median of SLE duration, demographic, clinical data and immunosuppressive therapy at the moment of the recruitment were obtained from the medical records. The following SLE-related factors were considered positive: ANA (immunofluorescence) > 1:80, *anti*-dsDNA antibodies (ELISA) > 15 U/ml and *anti*-ENA antibodies (ELISA) > 10 U/ml. Other lupus-related parameters or inflammation markers were determined, including erythrocyte sedimentation rate (ESR), complement fractions (C3, C4), high-sensitive C-reactive protein (hsCRP), plasma homocysteine levels, D-dimer (DD) and fibrinogen levels. Hypocomplementemia was considered when C3 and/or C4 fractions were below laboratory normal range while serological activity was defined as *anti*-dsDNA antibodies values above 15 U/ml and/or hypocomplementemia.

The activity of SLE was assessed by the SLE Disease Activity Index (SLEDAI), considering inactive disease when SLEDAI≤4 while organ damage was evaluated by the Systemic Lupus International Collaborative Clinics/American College of Rheumatology (SLICC/ACR) damage index, defining absence of organ damage when SLICC/ACR = 0.

### 2.2 Hemogram values

A fasting blood sample from each patient was obtained by venipuncture. Blood samples were tested with respect to leukocyte, lymphocyte, haemoglobin, mean corpuscular volume, platelet and RDW in our hospital haematology laboratory using an SYSMEX XN-20 automated haematology analyser (Roche, Basel, Switzerland). Neutrophil/lymphocyte, Platelet/lymphocyte and RDW/platelet ratios were directly calculated from the measured values. The reference range of RDW in our laboratory is 8%–14.8%, considering pathological values above 14.8%. Anemia was defined as a hemoglobin concentration value less than 12 g/dl.

### 2.3 Quantification of serum cytokine levels

Serum aliquots were obtained and stored at −80 °C until the measurement assays were carried out. The concentrations of serum inflammatory or anti-inflammatory cytokines IL-6, IL-10, Interferonγ (IFNγ) and Tumor necrosis Factor (TNF) were determined by flow cytometry using the CBA method (Cytometric Bead Array) (BD Bioscience, USA) which has a detection range of 2–5000 pg/ml. Sample processing was performed according to the manufacturer instructions. The samples were assessed with a FACScalibur flow cytometer and FCAP Array software was used for data analysis (BD Biosciences).

### 2.4 Statistical analysis

Quantitative variables were expressed as mean and standard deviation or as median and range, as appropriate; qualitative variables were expressed as frequency and percentage.

The Kolmogorov test was used to evaluate data distribution and as data did not follow a parametric distribution, statistical analysis was performed using Spearman rank's test to analyze correlations and Mann-Whitney *U* test to assess differences between groups. Levene's test was used for the homogeneity of variance test. The χ2 test (with the two-sided Fisher's exact test) was used to compare categorical variables. A lineal logistic regression was performed to determine the association between RDW and IL-6 considering age and hemoglobin as confounding factors. Finally, the discrimination ability was evaluated following an approach based on the area under the curve (AUC) - “receiver operating characteristic”- ROC. The Youden index, defined as the overall correct classification rate minus one at the optimal cut-off point, was added to determine which will be the value with the best sensitivity and specificity ratio to predict active disease. For all analyses, significance was defined as a P value of less than 0.05. Statistical analysis was performed using SPSS software.

## 3 Results

### 3.1 Subject characteristics

The clinical characteristics of patients included in the study, the features of the SLE patients, biochemical and immunological parameters and medications are summarized in Table 1. The mean disease duration was 13.5 years and ten patients (13%) met antiphospholipid syndrome criteria. Fourty-three patients (56%) had serological activity, based on the presence of positive antibodies *anti*-DNA and/or low complement levels. Nine patients (12%) were on no medication, 63 patients (81%) were taking antimalarials regularly either as the only treatment or in combination with some immunosuppressive drug. Five patients (7%) were receiving immunosuppressive therapy without antimalarials.

**Table 1:** Clinical characteristics and disease-specific features of SLE patients.

Age (years) (Median, range)	47 (19–80)
Disease duration (years) (Mean, SD)	13.5 (10.1)
Antiphospholipid syndrome (N,%)	10 (13)
Antimalarials only (N,%)	8 (10)
Antimalarials + Glucocorticoids (N,%)	21 (27)
Antimalarials + other immunosuppressive therapy (N,%)	39 (51)
No treatment (N,%)	9 (12)
Creatinine (<1.2 mg/dl) (Mean, SD)	0.8 (0.3)
D-dimer (0.1–0.5 μg/ml) (Mean, SD)	0.69 (2.02)
Fibrinogen (150–450 mg/dl) (Mean, SD)	355 (72)
ESR (0–13 mm) (Mean, SD)	19 (18)
hsCRP (0–10 mg/dl) (Mean, SD)	2.6 (5.3)
C3 complement (90.0–180.0 mg/dl) (Mean, SD)	104 (27)
C4 complement (10.0–40.0 mg/dl) (Mean, SD)	18 (9)
Positive ANA (>1:80) (N, %)	68 (88)
Positive *anti*-dsDNA antibodies (>15 U/ml) (N,%)	29 (38)
IL-6 (0.002–5 ng/ml) (Mean, SD)	4.42 (4.51)
Il-10 (0.002–5 ng/ml) (Mean, SD)	11 (49.44)
TNF (0.002–5 ng/ml) (Mean, SD)	2.69 (6.76
IFN (0.002–5 ng/ml) (Mean, SD)	2.26 (2.83)
Serological activity (N,%)	43 (56)
SLEDAI-2K (Mean, SD)	2.2 (2.5)
SLEDAI-2K ≥ 4 (N,%)	21 (27.3)
SLICC (Mean, SD)	1.8 (1.6)
SLICC ≥1 (N,%)	58 (75.3%)

ESR: Erythrocyte sedimentation rate, hsCRP: High-sensitive C-reactive protein, ANA: Antinuclear antibodies, *anti*-dsDNA: anti-double-stranded DNA antibody, TNF: Tumor necrosis factor, IFN: Interferon, SLEDAI: SLE Disease Activity Index, SLICC/ACR: Systemic Lupus International Collaborative Clinics/American College of Rheumatology.

The 80 healthy women in the control group have a median age of 42 years (range 18–64).

### 3.2 Hematological parameters in SLE patients and healthy donors

Hematological parameters and ratios for all SLE patients, those for SLE with anemia (SLE-a), SLE without anemia (SLE-na) and healthy donors are compared in Table 2. SLE patients presented significantly higher RDW, NLR, PLR and lower lymphocyte counts than the control group (p < 0.001). Considering anemia status, SLE patients without anemia had higher RDW values when compared to healthy donors (p < 0.02) while SLE patients with anemia (SLE-a) showed significantly higher RDW, NLR and PLR values, as compared to patients without anemia (SLE-na) (p < 0.0001).

**Table 2:** Blood count values in SLE and healthy donors.

	SLE (n = 77)	SLE-na (n = 63)	SLE-a (n = 14)	Controls (n = 80)
Age (Median, range)	47 (19–80)	45 (19–80)	48 (36–64)	42 (18–64)
Hemoglobin (g/dl), (Mean, SD)	13.12 ± 1.31	13.59 ± 0,84	10.99 ± 0,88**	13.79 ± 0,87
Hematocrit (%), (Mean, SD)	39.50 ± 3,48	40.68 ± 2,37	34.27 ± 2,80**	42.32 ± 2,68
MCV (%), (Mean, SD)	87.62 ± 4,15	87.87 ± 3,90	86.51 ± 5,18**	93.15 ± 5.36
MCH (pg), (Mean, SD)	32.95 ± 1.73	33.26 ± 1.39	34.21 ± 2.42	32.61 ± 1.13
RDW (%), (Mean, SD)	13.69 ± 1.48*	13.52 ± 1.36*^†^	14.51 ± 1.79*^#^	12.89 ± 0.93
NLratio (NLR), (Mean, SD)	2.11 ± 1.06**	1.99 ± 1.08	2.65 ± 0.81^**#**^	1.38 ± 0.62
PLratio (PLR), (Mean, SD)	164.60 ± 73.11**	154.95 ± 67.90	208.01 ± 82.36^**#**^	98.85 ± 26.92
Lymphocytes (10^3^/μl), Mean, SD)	1.53 ± 0.70**	1.55 ± 0.54**	1.45 ± 0.55**	2.72 ± 0.79
Neutrophils (10^3^/μl), (Mean, SD)	2.99 ± 0.15^**†**^	2.81 ± 0.14^†^	3.78 ± 0.18	3.52 ± 0.13
Platelets (10^3^/μl), (Mean, SD)	224.9 ± 63.4	215.2 ± 61.2	268.4 ± 56.3	253.9 ± 54

* Compared to controls, p < 0.001; ** Compared to controls, p < 0.0001; #: Compared to SLE-na, p < 0.0001; †: Compared to controls, p < 0.05.

SLE-na: SLE without anemia, SLE-a: SLE with anemia, MCV: mean corpuscular volume, MCH: mean corpuscular hemoglobin, RDW: Red blood cell distribution width.

### 3.3 RDW, NLR and PLR association and correlation with clinical parameters, disease activity and SLE treatment

Classical inflammation markers, SLE disease markers, SLEDAI, SLICC/ACR, treatment received by patients, and their relationship with RDW, NLR and PLR were analyzed.

The three parameters were significantly associated with the presence of hypocomplementemia (p < 0.05) (Table 3). PLR value was significantly associated with low C3 complement and C4 complement levels (p<0.05), and showed a significant increase (p = 0.020) in the group of patients with active disease measured by SLEDAI-2K score. Furthermore, PLR correlated with the disease duration (p < 0.05). Similarly, both NLR and PLR showed a significant inverse correlation with C3 complement level (p < 0.02).

**Table 3:** Association between RDW, NLR and PLR and clinical activity parameters.

	RDW			NLR			PLR		
	Presence	Absence	p	Presence	Absence	p	Presence	Absence	p
ANA	13.68 ± 1.88	13.77 ± 0.92	0.339	2.06 ± 1.06	2.24 ± 0.83	0.416	160.38 ± 73.45	178.46 ± 40.18	0.223
Anti dsDNA antibodies	13.73 ± 1.56	13.66 ± 1.46	0.831	1.82 ± 0.71	2.24 ± 1.17	0.217	164.38 ± 73.07	161.00 ± 69.99	0.843
Antiphospholipid syndrome	14.17 ± 2.04	13.78 ± 1.62	0.530	2.29 ± 0.98	2.14 ± 1.17	0.446	149.68 ± 104.66	176.43 ± 78.72	0.143
Hypocomplementemia	14.04 ± 1.54	13.51 ± 1.43	**0.035**	2.31 ± 0.84	2.00 ± 1.16	**0.046**	182.21 ± 66.70	155.08 ± 75.28	**0.038**
Low C3	14.17 ± 1.74	13.53 ± 1.36	0.080	2.31 ± 0.76	2.04 ± 1.15	0.083	190.59 ± 68.78	155.48 ± 72.96	**0.017**
Low C4	13.88 ± 1.54	13.65 ± 1.48	0.322	2.29 ± 0.86	2.06 ± 1.11	0.187	200.72 ± 76.37	155.86 ± 70.16	**0.027**
Serological activity	14.35 ± 1.66	13.55 ± 1.43	**0.047**	1.96 ± 0.64	2.10 ± 1.10	0.667	187.70 ± 56.55	157.05 ± 72.57	0.062
Disease activity (SLEDAI-2K)	14.12 ± 1.87	16.62 ± 1.40	0.278	2.53 ± 1.27	2.04 ± 1.02	0.132	208.87 ± 84.46	157.12 ± 68.33	**0.020**
Organ damage (SLICC/ACR)	13.77 ± 1.48	13.48 ± 1.49	0.372	2.15 ± 1.857	1.98 ± 0.75	0.822	163.57 ± 72.19	167.71 ± 77.80	0.662
Current treatment for SLE	13.74 ± 1.53	13.38 ± 1.11	0.494	2.18 ± 1.10	1.57 ± 0.45	**0.006**	167.28 ± 76.56	144.34 ± 34.16	0.380
Corticosteroids	14.04 ± 1.61	13.31 ± 1.23	**0.030**	2.21 ± 1.21	1.99 ± 0.86	0.351	173.73 ± 80.98	154.19 ± 62.47	0.244
Hydroxychloroquine	13.76 ± 1.57	13.40 ± 0.94	0.409	2.14 ± 1.12	1.95 ± 0.81	0.539	166.87 ± 78.62	154.36 ± 40.37	0.566

RDW: Red blood cell distribution width, PLR: Platelet-to-lymphocyte ratio, NLR: Neutrophil-to-lymphocyte ratio, ANA: Antinuclear antibodies, APS: Antiphopholipid syndrome, SLEDAI: SLE Disease Activity Index, SLICC/ACR: Systemic Lupus International Collaborative Clinics/American College of Rheumatolog

RDW was associated with the serological activity of the patients (p < 0.05) and was positively correlated with SLEDAI-2K and SLICC/ACR scores, hsCRP, D-dimer, fibrinogen, IL-6 and TNF (p < 0.05, for all) (Table 4). No association was observed with the antibody profile.

**Table 4:** Correlations between RDW, NLR and PLR and clinical parameters.

	RDW		NLR		PLR	
	R	p	r	p	r	p
Disease duration (years)	0.143	0.213	0.247	0.030	0.181	0.114
SLEDAI-2K	0.288	**0.012**	0.111	0.343	0.183	0.116
SLICC/ACR	0.235	**0.039**	0.017	0.881	−0.072	0.531
C3 complement (mg/dl)	−0.165	0.153	−0.249	**0.019**	−0.324	**0.004**
C4 complement (mg/dl)	0.055	0.637	−0.150	0.194	−0.140	0.224
ESR (mm/h)	0.041	0.732	0.094	0.436	0.039	0.746
hsCRP (mg/l)	0.258	**0.026**	0.094	0.426	−0.154	0.190
Fibrinogen (mg/dl)	0.255	**0.025**	−0.022	0.852	0.051	0.659
Homocysteine (μmol/L)	0.193	0.097	0.061	0.605	0.016	0.890
Ddimer (0.23 μg/ml)	0.238	**0.039**	0.160	0.166	0.161	0.164
Creatinine (mg/dl)	0.117	0.312	−0.080	0.489	−0.181	0.115
Creatinine clearance (Cockcroft)	−0.131	0.262	0.060	0.612	−0.009	0.940
IL-6 (pg/ml)	0.325	**0.012**	0.101	0.445	0.065	0.624
IL-10 (pg/ml)	0.119	0.368	0.065	0.624	−0.101	0.445
TNF (pg/ml)	0.260	**0.047**	0.036	0.787	0.091	0.543
IFNγ (pg/ml)	0.128	0.342	0.007	0.959	0.247	0.064

RDW: Red blood cell distribution width, NLR: Neutrophil-to-lymphocyte ratio, PLR: Platelet-to-lymphocyte ratio, SLEDAI: SLE Disease Activity Index, SLICC/ACR: Systemic Lupus International Collaborative Clinics/American College of Rheumatology, ESR: Erythrocyte sedimentation rate, hsCRP: High-sensitive C-reactive protein, TNF: Tumor necrosis factor, IFNγ: Interferon γ

With regard to the treatment received for their disease, patients with active treatment exhibited a significantly higher NLR (p=0.006), and their RDW value was significantly associated with corticosteroids intake (p = 0.030), while no associations were observed with the antimalarial treatment.

Considering anemia, the group of SLE-a patients showed a strong correlation of RDW with serum IL-6 (r = 0.894, p < 0.001) in addition to PLR with TNF (r = 0.743, p < 0.025) and an inverse correlation with C3 complement (r = 0.538, p < 0.05). Furthermore, most of these associations were maintained in the SLE-na group, including C reactive protein, hypocomplementemia, IL-6, SLEDAI and steroid use.

Finally, a linear logistic regression analysis confirmed that, after adjustment by age and hemoglobin values, RDW presented linear correlation with IL-6 levels (Beta-coefficient = 0.369, p = 0.003).

### 3.4 ROC curves for hemogram values and disease activity

Receiver-operating curves (ROC) were performed to differentiate between active and inactive SLE patients, considering SLEDAI-2K ≥ 4 or hypocomplementemia to define active SLE. The results are shown in Table 5. For both SLEDAI-2K ≥ 4 and hypocomplementemia, PLR showed better discrimination ability.

**Table 5:** RDW, NLR and PLR AUC-ROC analysis for active disease.

	SLEDAI-2K ≥ 4				Hypocomplementemia			
	AUC-ROC	Cut-off	S	1-E	AUC-ROC	Cut-off	S	1-E
RDW	0.561	13.93	0.46	0.24	0.646	13.21	0.74	0.40
NLR	0.630	1.97	0.69	0.40	0.639	1.68	0.74	0.38
PLR	0.680	154	0.77	0.47	0.644	127	0.85	0.56

AUC-ROC: Area Under the Curve - Receiver Operating Characteristic. S: Sensitivity, 1-E: Especificity, RDW: Red blood cell distribution width, PLR: Platelet-to-lymphocyte ratio, NLR: Neutrophil-to-lymphocyte ratio.

* The table shows the discrimination ability analysis of the three parameters by the AUC-ROC. The Youden index or cut-off point determines which will be the value with the best sensitivity (S) and specificity ratio (1-E) to predict active disease.

## 4 Discussion

Our results indicate that RDW, NLR and PLR are higher in SLE patients compared to healthy controls. Their values also suggest active disease as they are higher in the group of SLE with anemia and those with lower complement levels, higher inflammatory markers, certain cytokines associated with SLE physiopathology and in patients with higher SLEDAI and SLICC scores.

In our cohort, both NLR and PLR were higher in SLE than in controls. Furthermore, both parameters showed negative correlation with haemoglobin and hypocomplementemia (C3 and C4) while PLR exhibited a significant increase in the group of patients with active disease measured by SLEDAI and was correlated with disease duration. Our results coincide with that previously described when NLR and PLR have been studied in SLE and other autoimmune rheumatic diseases [[6], [7], [8]]. These findings highlight the central role of the lymphocyte and lymphopenia in SLE physiopathology over neutropenia or thrombopenia, common in this context and observed in our cohort [15].

In regard to RDW, it is a readily available parameter that have shown that could be an interesting tool to assess SLE disease activity since it has been associated with C3, C4, CRP, ESR, IgM, SLEDAI-2K score [[6], [7], [8], [9], [10], [11]] or even to certain symptoms such as fatigue [12]^.^ Our study not only confirms these results measured in the same cohort but also adds interesting findings.

Firstly, RDW was higher in SLE patients than in controls, irrespective of anemia status, highlighting the role of SLE itself on red cell damage and the inflammatory environment. Thus, RDW was associated with serological activity, inflammatory markers such as CRP, fibrinogen or D-dimer in addition to SLEDAI-2K score. However, not previously reported, RDW correlated with SLICC, an SLE index score that has been associated with disease duration, number of flares and use of glucocorticoids [15]. This finding supports RDW being a marker of chronic inflammation and tissue damage.

Secondly, RDW showed an interesting correlation with IL-6 and TNF-alpha. Moreover, the correlation with IL-6 was very strong in patients with anemia who were also more active. On the one hand, IL-6 participates in T-cell differentiation, B-cell maturation, synthesis and the secretion of immunoglobulins and is one of the main cytokines underlying the anemia of chronic disease [16,17]^.^ In addition to diminished response to erythropoietin and reduced erythrocyte survival, IL-6 inhibits erythropoiesis and hemoglobin synthesis in the bone marrow. On the other hand, TNF-alpha is a key cytokine of the immune system that induces several chemokines, endothelial adhesion molecules (ICAM-1, VCAM-1 …), inflammatory cytokines (including IL-6) and also enhances leukocyte recruitment and migration [18]. Thus, it has a key role in systemic inflammation; TNF-alpha also has an essential role in anemia of chronic disease through stimulating ferritin synthesis, degradation and phagocytosis of red cells and direct inhibition of erythropoiesis (iron-restricted) [16,17]. Both IL-6 and TNF-alpha cytokines have been shown to have an important role in SLE. Firstly, IL-6 has been found to be correlated with ESR, *anti*-dsDNA antibodies, disease activity, and anemia [[19], [20], [21], [22], [23], [24]]. In parallel, TNF-alpha has been shown to be higher in patients with active disease, higher SLEDAI and hematological disease [[25], [26], [27]].

Therefore, since RDW reflects the variation in the size of erythrocytes secondary to altered erythropoiesis and blockage of hemoglobin synthesis in the bone marrow, our results support RDW possibly being a marker of the effect of IL-6 and TNF-alpha on iron metabolism and bone marrow. Similar findings have been described in diverse settings such as heart disease or cardiovascular disease, sepsis and RA [[28], [29], [30]] but, to our knowledge, the correlation with RDW and these cytokines has not been described in SLE.

Moreover, no association was observed between these parameters and antimalarial or immunosuppressive treatments except for RDW and corticoids. While Hu et al. demonstrated that RDW levels decreased with steroid treatment, in our cohort higher RDW was seen in patients who were receiving steroids [10]. However, our group of patients receiving steroids had otherwise higher IL-6 and IFN, and, without statistical difference, tend to have higher DNA antibodies, SLEDAI, SLICC and lower complement. Thus, our data suggests that there were probably more active patients and therefore with higher indication of steroid treatment.

Nevertheless, the limitations of this study have to be considered. In addition to being a single-center, observational and retrospective study with a small population size; the fact that there were mainly stable patients with little inflammatory activity and long-standing disease could have reduced our ability to find associations between markers and serological or clinical activity. In addition, and due to population size, we were not able to evaluate these parameters in non-treated SLE patients, or considering the role of certain drugs such as glucocorticoids, antimalarials or immunosuppressants. Finally, neither the cause of the anemia or other concomitant conditions such as iron-deficiency anemia, anemia related to chronic kidney disease or hemolytic anemia were evaluated. These factors could have been modifiers of the hemogram parameters. Despite this, our cohort represents a real life and heterogeneous cohort and therefore, we consider that our data are generalizable and can be extrapolated to real clinical practice.

In conclusion, NLR, PLR and RDW are parameters related to SLE that suggest SLE serological and clinical activity. Given their availability, these markers could be useful tools to identify and monitor active SLE patients and therefore candidates for close follow-up or eventual immunosuppressive treatment. In addition, these parameters could be considered in inflammatory pathologies led by IL-6 and TNF pleiotropic effects. Further studies are needed, with a greater number of patients and at different stages of activity, to validate this association and clarify the value of NLR, PLR and RDW as markers of lupus activity or response to treatment.

## Credit author statement

Víctor Moreno-Torres: Conceptualization, Methodology, Software, Validation, Formal Analysis, Investigation, Resources, Data curation, Writing-original draft, Writing-review and editing, Visualization., Raquel Castejon: Conceptualization, Methodology, Software, Validation, Formal Analysis, Investigation, Resources, Data curation, Writing-original draft, Writing-review and editing, Visualization, Supervision, Project administration, Funding acquisition., Susana Mellor-Pita: Investigation, Resources, Writing-original draft, Writing-review and editing, Supervision., Pablo Tutor-Ureta: Investigation, Resources, Writing-original draft, Writing-review and editing., Pedro Durán-del Campo: Investigation, Resources, Writing-original draft, Writing-review and editing., María Martínez-Urbistondo: Conceptualization, Methodology, Software, Formal Analysis, Investigation, Data curation, Writing-original draft, Writing-review and editing, Visualization., José Vázquez-Comendador: Conceptualization, Methodology, Software, Formal Analysis, Investigation, Data curation, Writing-original draft, Writing-review and editing, Visualization., Ángela Gutiérrez-Rojas: Conceptualization, Methodology, Software, Formal Analysis, Investigation, Data curation, Writing-original draft, Writing-review and editing, Visualization., Silvia Rosado: Methodology, Software, Validation, Resources, Data curation, Writing-original draft, Writing-review and editing, Project administration, Funding acquisition., Juan A Vargas-Núñez: Conceptualization, Investigation, Resources, Writing-original draft, Writing-review and editing, Visualization, Supervision, Project administration, Funding acquisition.

## Declaration of competing interest

The authors declare that they have no known competing financial interests or personal relationships that could have appeared to influence the work reported in this paper.
